# Arsenic mobilization in shallow aquifers due to CO_2_ and brine intrusion from storage reservoirs

**DOI:** 10.1038/s41598-017-02849-z

**Published:** 2017-06-05

**Authors:** Ting Xiao, Zhenxue Dai, Hari Viswanathan, Alexandra Hakala, Martha Cather, Wei Jia, Yongchao Zhang, Brian McPherson

**Affiliations:** 1 Earth and Environmental Sciences Division, Los Alamos National Laboratory, Los Alamos, New Mexico 87545 USA; 2 Department of Civil and Environmental Engineering, University of Utah, Salt Lake City, UT 84112 USA; 3 Energy and Geoscience Institute, University of Utah, Salt Lake City, UT 84108 USA; 4 College of Construction Engineering, Jilin University, Changchun, 130026 China; 5 Key Laboratory of Groundwater Resources and Environment, Ministry of Education, Jilin University, Changchun, 130021 China; 6 U.S. Department of Energy, National Energy and Technology Laboratory, Pittsburgh, PA 10940 USA; 7 Petroleum Recovery Research Center, New Mexico Institute of Mining and Technology, Socorro, NM 87801 USA; 8 College of Geosciences, China University of Petroleum, Beijing, 102249 China

## Abstract

We developed an integrated framework of combined batch experiments and reactive transport simulations to quantify water-rock-CO_2_ interactions and arsenic (As) mobilization responses to CO_2_ and/or saline water leakage into USDWs. Experimental and simulation results suggest that when CO_2_ is introduced, pH drops immediately that initiates release of As from clay minerals. Calcite dissolution can increase pH slightly and cause As re-adsorption. Thus, the mineralogy of the USDW is ultimately a determining factor of arsenic fate and transport. Salient results suggest that: (1) As desorption/adsorption from/onto clay minerals is the major reaction controlling its mobilization, and clay minerals could mitigate As mobilization with surface complexation reactions; (2) dissolution of available calcite plays a critical role in buffering pH; (3) high salinity in general hinders As release from minerals; and (4) the magnitude and quantitative uncertainty of As mobilization are predicated on the values of reaction rates and surface area of calcite, adsorption surface areas and equilibrium constants of clay minerals, and cation exchange capacity. Results of this study are intended to improve ability to quantify risks associated with potential leakage of reservoir fluids into shallow aquifers, in particular the possible environmental impacts of As mobilization at carbon sequestration sites.

## Introduction

Geologic CO_2_ sequestration (GCS) is considered a promising approach for mitigating CO_2_ emissions from centralized sources^1–9^. One major concern is the risk of CO_2_ and/or brine leakage from deep sequestration reservoirs through highly-permeable zones such as faults and abandoned wells into overlying underground sources of drinking water (USDW)^10^. Carbon dioxide itself is not hazardous to water quality, but increased CO_2_ concentrations in shallow groundwater aquifers could reduce pH and enhance geochemical reactions between groundwater and aquifer sediments, resulting in release and mobilization of toxic trace metals^11, 12^. An additional risk is the leakage of reservoir brine, which may contain toxic substances, into USDWs^13, 14^.

To assess the risk of CO_2_/brine leakage to overlying USDWs and to detect signatures of aquifer quality changes at early stages, various approaches have been conducted with lab-scale experiments^11, 15–17^, short-term field-scale tests^18–20^, numerical modeling^21–27^, and natural analog observations^13, 28^. Most of these studies focus on CO_2_ leakage and its impacts on groundwater quality, but a limited number of studies have examined the leakage of brine with/without CO_2_ into shallow aquifers. Keating *et al*.^29^ observed the upward migration of CO_2_ and saline water under natural conditions, which affected the salinity and trace metal concentrations in shallow groundwater. To date, modeling approaches combined with laboratory/field observations are necessary for studies of the geochemical impacts of leaked CO_2_ in shallow aquifers, to reduce the uncertainties of modeling itself and to interpret the observation data with appropriate reaction patterns. Yang *et al*.^27^ developed an inverse multicomponent geochemical modeling approach to interpret responses of water chemistry to the introduction of CO_2_ into a set of laboratory batch reactors containing carbonate-poor and carbonate-rich potable aquifer sediments. Bacon *et al*.^21^ applied multiphase reactive transport modeling to identify potential trace metal release mechanisms under elevated CO_2_ conditions in a carbonate aquifer with both batch and column experiments. However, limited studies have been conducted with such combined approaches for geochemical mechanisms under saline conditions to interpret the responses to brine leakage, which is also significant for quantitative risk analysis of GCS projects.

The primary goal of this paper is to elucidate the mechanisms of trace metal mobilization with an integrated experimental and simulation framework. The case study includes elevated CO_2_ conditions at the Chimayo site in northern New Mexico, a natural analog with CO_2_ upwelling. Arsenic (As) is relatively rich within the sediments and is a potential source of high As concentration in local water, thus As mobilization is of specific interest. Batch experiments of water-rock-CO_2_ interactions are conducted with both fresh groundwater and saline water, to mimic scenarios of CO_2_ leakage with and without deeper formation brine. Quantitative interpretation of As mobilization due to leaked CO_2_ and brine utilizes an inverse reactive transport modeling approach. A specific objective for this study is to identify the responses of As release under different water salinities and to quantify the key parameters controlling As mobilization processes.

## Results

### Water chemistry changes

**Figure 1:**
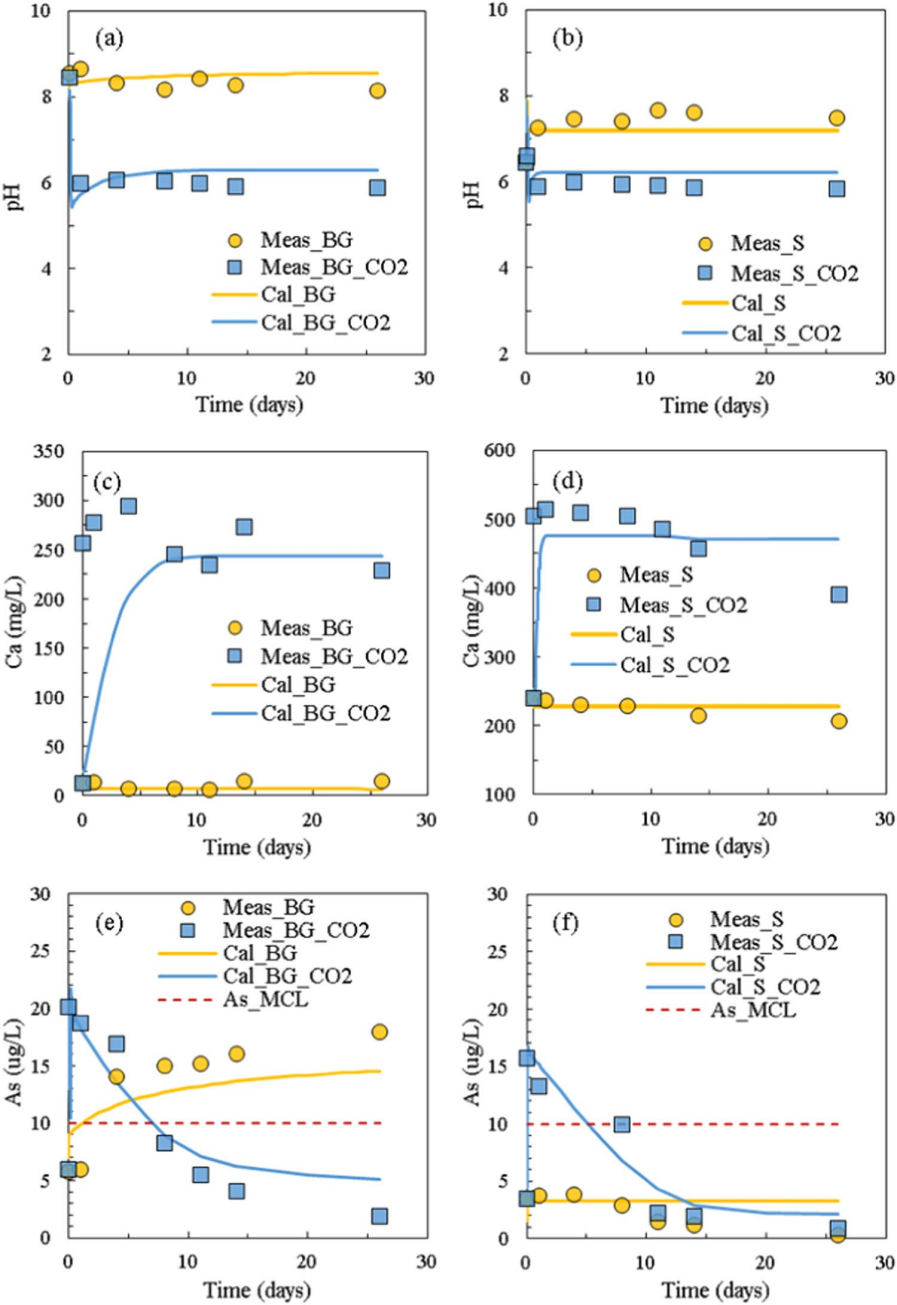
Measured (symbols) and calculated (lines) water chemistry of the batch experiments: (**a**) background (BG, TDS ~200 mg/L) pH; (**b**) saline (S, TDS ~4000 mg/L) pH; (**c**) background Ca; (**d**) saline Ca; (**e**) background As; (**f**) saline As. Maximum contaminant level (MCL) of As is also marked in (**e**) and (**f**).

When CO_2_ is introduced into the water-rock batch systems, pH decreases immediately because of CO_2_ dissolution in both background (BG) and saline (S) samples (Fig. 1a,b). After the sharp drop, pH remains stable at ~5.9, even after CO_2_ injection is stopped. The simulated pH also shows a sudden drop within 1 h of CO_2_ injection, then a slight increase, and finally reaches a steady-state. This phenomenon was also observed by Shao *et al*.^17^ and Yang *et al*.^27^. This process is mainly affected by CO_2_ diffusion and mineral reactions, and the dissolution of minerals (especially calcite) consumes hydrogen ions and causes the solution pH to increase slightly between 4 and 72 h^17^. For the CO_2_-free (control) experiments, both measured and calculated pH remain stable at ~8.3 for BG and ~7.3 for S (Fig. 1a,b), and good matches with high confidence are achieved between calculated and experimental results.

Synthetic groundwater and saline water were used in our experiments, and the fast dissolving minerals (such as calcite) are not abundant in the system. This makes it hard to track major ion concentration changes in the water samples during the experiments, especially for Mg, Na, Si, Cl and SO_4_ in the saline-water reactor when CO_2_ is introduced. Only Ca shows a more than 50% concentration increase compared to the CO_2_-free experiments, indicating that calcium minerals (especially calcite) dissolved during the experiments (Fig. 1c,d).

With CO_2_ intrusion into the shallow groundwater aquifer, trace metals of environmental concern might be released. Arsenic is of specific interest in our study due to its high concentration in the shallow groundwater of the Chimayo site^13^. Figure 1e–f shows As concentration changes of the batch experiments and simulated results. The simulated results match well with the experimental measurements, which capture the trends of As concentration changes in all four cases. Without CO_2_ introduction, it shows an increase of As concentration and reaches to equilibrium after sediment and water mixed for both BG and S systems. When CO_2_ is introduced to the reactors, it shows a sharp increase of As concentration at the initial time, suggesting a large amount of As released from the sediments due to CO_2_ intrusion. After a few hours, As concentration starts to decrease slowly, and reaches equilibrium after 26 days. For both reactors with and without CO_2_ introduction, the BG reactors show higher increases of As concentrations than that in the S reactors (20.1 µg/L vs. 15.7 µg/L for the sharp increase with CO_2_ introduction and 17.9 µg/L vs. 3.8 µg/L of the control reactors), which indicates that salinity impacts the behavior of As mobilization. One possible reason is that high aqueous salinity reduces the dissolution of minerals (e.g. calcite and clay minerals), affects the water-rock equilibrium and system pH, which further hinders As release. Although As concentration exceeds the maximum contamination level (MCL) of the U.S. Environmental Protection Agency (EPA) in the beginning when CO_2_ is introduced for both BG and S reactors, it drops below the MCL after 8-day exposure, which might not be considered as a long term concern of the USDWs. This As concentration drop was also observed in other studies^17, 30, 31^. It is notable that the BG reactor without CO_2_ injection shows a large increase of aqueous phase As concentration to exceed the MCL. However, the field samples show low concentrations of As (~1 µg/L) without CO_2_ and brine exposure^13^. This reveals the limitation of batch experiments in that they overestimate the water-rock and water-rock-CO_2_ reactions within well-mixed water-sediment systems and large reaction areas of mineral surface, which is also pointed out by other researchers^18, 27^.

### Geochemical reaction parameters

The inverse modeling approach is applied for estimating geochemical reaction parameters for each experiment in this study. Estimated mean values of geochemical reaction parameters from the four experiments are shown in Table 1. The coefficient of variation (CV, the ratio of the standard deviation to the mean value) is also shown in the table to suggest the extent of variability of each parameter. Generally, the estimated values are in agreement with those reported by literature^12, 25, 32, 33^, which suggests high confidence of the parameter estimation approach. However, the parameters with large CV values indicate high uncertainties among different specific cases. Especially, mineral reaction rates and mineral surface areas of calcite and clay minerals vary with up to two orders of magnitudes among the four cases, indicating that these parameters should be carefully chosen for simulations, and our estimation can provide a reference for determining the range of these parameters. To verify that the estimated mean values could represent the scenarios of all the cases, the model was simulated with these mean values for all the four experiments. The results indicate that these parameters are reasonable for geochemical reaction simulations especially for the analysis of As behaviors with/without CO_2_ introduction under a large range of salinity conditions.

**Table 1:** Estimated geochemical reaction parameters from the batch experiments.

Category	Name	Symbol	Estimated mean value	CV
Mineral dissolution/precipitation rate constant (mol/m^2^/s)	Calcite dissolution	cal_rkf1	3.52 × 10^−6^	0.92
	Calcite precipitation	cal_rkf2	1.42 × 10^−6^	0.69
	Kaolinite	kao_rkf	1.06 × 10^−12^	1.17
	Illite	ill_rkf	1.18 × 10^−12^	0.70
	Smectite	sme_rkf	8.57 × 10^−12^	1.23
	Hematite	hem_rkf	1.45 × 10^−12^	0.87
	K^−^feldspar	fel_rkf	1.39 × 10^−10^	1.33
	Quartz	qua_rkf	1.94 × 10^−14^	0.15
	Albite	alb_rkf	2.70 × 10^−12^	0.26
	Anorthite	ano_rkf	1.04 × 10^−14^	0.97
Mineral surface area (cm^2^/g)	Calcite	cal_amin	53.96	0.98
	Kaolinite	kao_amin	316.84	1.15
	Illite	ill_amin	272.06	1.11
	Smectite	sme_amin	24.90	1.37
	Hematite	hem_amin	274.08	0.43
	K-feldspar	fel_amin	222.42	1.87
	Quartz	qua_amin	23.29	0.24
	Albite	alb_amin	27.48	1.00
	Anorthite	ano_amin	257.60	1.92
Adsorption surface area (cm^2^/g)	Hematite-OH	hem_soh_ssa	2.13	0.62
	Kaolinite-OH	kao_soh_ssa	11018.86	0.95
	Illite-OH	ill_soh_ssa	2.82	0.86
	Smectite-OH	sme_soh_ssa	65.83	0.75
Surface complex equilibrium constant (logK)	(Hematite)_2_−AsO4^−^	logK_hem	−9.17	0.22
	Illite- HAsO4^−^	logK_ill	−10.28	0.01
	Smectite-HAsO4^−^	logK_sme	3.94	0.30
	Kaolinite-AsO_4_^2−^	logK_kao	1.15	0.59
Cation exchange capacity (meq/100 g)	CEC	cec	2.92	0.54
Cation exchange selectivity	KNa/H	h_ekx	0.202	0.01
	KNa/Ca	ca_ekx	0.748	0.27
	KNa/Mg	mg_ekx	4.19 × 10^−4^	0.45
	KNa/K	k_ekx	2.13 × 10^−2^	0.23

**Figure 2:**
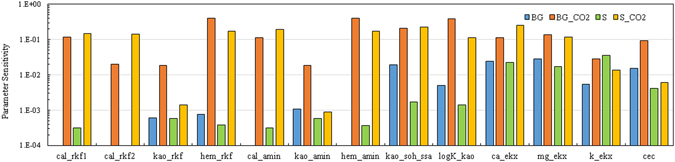
Sensitivity analysis of the geochemical reactive parameters.

A composite sensitivity analysis of the geochemical parameters was conducted to explore the most sensitive parameters to the experimental results by calculating their composite sensitivity coefficients^34, 35^. The most sensitive parameters are listed in Fig. 2. Generally, Sensitivity is increased when CO_2_ is introduced, indicating that the related reactions are pH dependent. With CO_2_ introduction, the model is highly sensitive to the calcite reaction rates (cal_rkf1 and cal_rkf2), hematite dissolution rate (hem_rkf), surface areas of calcite (cal_amin) and hematite (hem_amin), kaolinite adsorption surface area (kao_soh_ssa), and kaolinite adsorption equilibrium constant (logK_kao). The cation exchange selectivity and CEC showed relatively high sensitivities for all the four cases in our study, indicating that cation exchange reactions are significant for aqueous phase ion concentrations including major ions (Ca, Mg, Na, K) and trace metals (As).

Overall, the inverse modeling approaches provide reasonable geochemical reactions for our study, suggesting that it is possible to predict groundwater chemistry responses with CO_2_ intrusion in other cases. However, there are still some limitations with such approaches. For example, the range and initial value of key parameters should be carefully chosen to obtain a reasonable outcome; there might be more than one solution leading to a good representation of the observation data; and uncertainties of the model structure and observation data could also impact the estimations^36^. Therefore, experimental studies of mechanisms of geochemical reactions are meaningful to provide reasonable reaction pathways and the ranges of key parameters under different conditions. Our study using the combined application of batch experiments with geochemical simulation approaches can provide a good example for future research with multiple reaction parameters for CO_2_ and/or brine leakage studies.

## Discussion

### Arsenic mobilization mechanisms

**Figure 3:**
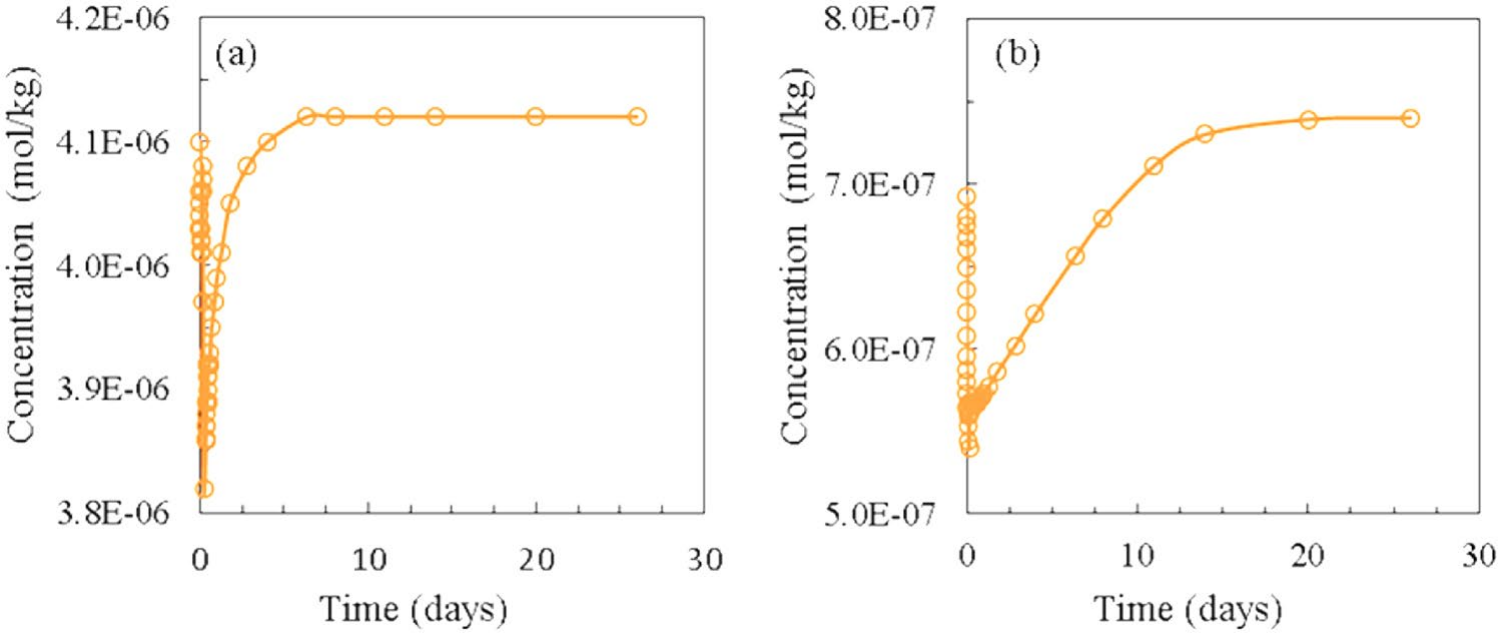
Simulated adsorbed As on kaolinite with CO_2_ introduction: (**a**) Background; (**b**) Saline.

Arsenic mobilization behavior has been widely discussed by researchers because it is one of the major concerns for groundwater quality in the event of CO_2_ leakage. Most of the studies consider the behavior of As to be largely related to adsorption/desorption onto/from the surfaces of clay and Fe-rich minerals^30, 37, 38^, and some researchers believe that As-bond mineral dissolution (i.e. As-carbonate minerals and Arsenopyrite) is the primary source of As contamination^12, 39, 40^. The two mechanisms are sometimes combined to explain As mobilization behavior in experimental and modeling approaches^12, 25^. It is hard to quantitatively determine the formula and fractions of such As-bond minerals because of their low concentrations, and estimated formula and volume fractions are usually used in simulations^12^. To verify As mobilization mechanisms and to demonstrate the role of As mineral dissolution and surface adsorption/desorption, a small volume fraction (<0.01%) of As-calcite (CaCO_3_·As_2_O_5_) was added to the numerical model as an uncertainty parameter to analyze its impact to As behavior. The results show that the sensitivity of As concentrations to such mineral dissolution is far less than that to surface adsorption/desorption process, and As-calcite dissolution shows minimal impact on As concentration in the aqueous phase. The reason is because the mineral dissolution rate is far less than surface complexation processes, and its dissolution is limited during the experiments. According to our simulation results, adsorption/desorption onto/from the surfaces of clays, especially kaolinite, controls As mobilization with water-rock-CO_2_ interactions. Arsenic also shows adsorption/desorption to illite, hematite and smectite, but with a few orders of magnitude less than that adsorbed on kaolinite. Figure 3 shows the simulated adsorbed As on kaolinite with CO_2_ introduction to both BG and S reactors. With CO_2_ introduction, there is an immediate sharp drop of adsorbed As, and it slowly re-adsorbs to local equilibrium in 7–15 days afterwards. The surface complexation reactions of the clay minerals (S represents mineral sites) could be written as^41^:

1 $$SO{H}_{(s)}+{H}_{(aq)}^{+}\rightleftharpoons SO{H}_{2(s)}^{+}$$

2 $$SO{H}_{(s)}\rightleftharpoons S{O}_{(s)}^{-}+{H}_{(aq)}^{+}$$

3 $$SO{H}_{(s)}+{H}_{3}As{O}_{4(aq)}\rightleftharpoons SAs{O}_{4(s)}^{2-}+2{H}_{(aq)}^{+}+{H}_{2}O$$

The mass balance for the total numbers of reactive sites (omit other adsorbed metals) is:

4 $${[SOH]}_{T}=[SOH]+[SO{H}_{2}^{+}]+[S{O}^{-}]+[SAs{O}_{4}^{2-}]$$

[SOH]_T_ is related to the adsorption site density and mineral volume fraction, and the surface functional groups are competitive for the available sites. During the experimental time in our study, clay minerals do not show significant volume change, thus we can assume [SOH]_T_ to be a constant value for this discussion. With the sudden drop of pH (increase of H^+^) at the initial time, it promotes reaction (2), and SOH_2_^+^ rapidly occupies the surface complexation sites, which results in As release from SAsO_4_^2−^ to the aqueous phase (H_3_AsO^4^). With the buffering effect of calcite dissolution (CaCO_3_ + H^+^ ⇌ Ca^2+^  + HCO_3_^−^), the pH of the system starts to increase slowly to a certain extent (Fig. 1a,b), and a series of local equilibriums are reached with SAsO_4_^2−^/H_3_AsO_4_ for gradual As adsorption onto clay minerals (Equation (3)). Without CO_2_ introduction, because of the low As concentration in aqueous phase, the adsorbed As releases from the clay mineral adsorption sites to reach to a series of local equilibriums, which results in an increase in As concentration in the water (Fig. 1e,f).

### Environmental implications

Trace metal contamination, especially As, is always a major concern when considering the potential risks of CO_2_ and saline water leakage from GCS sites. Results of this study suggest that, in general, As may be considered an insignificant long-term concern in a CO_2_ rich environment because of clay adsorptions. Likewise, in a saline environment, high concentrations of major ions (Ca, Mg, Na *et al*.) could hinder As release from the clay mineral sites. In such circumstances, if As is present, the reservoir brine might contain low concentrations of As and other trace metals to begin with (i.e. not due to enhanced desorption and mobilization). In many cases, increased salinity of USDW via the leaked saline water may likely be a larger concern than any associated released the trace metals^42^. Observed data from the Chimayo site suggest that the brackish water leaked through faults provides a source of arsenic and other heavy metals^13^. Kirsh *et al*.^39^ and Karamalidis *et al*.^40^ showed As concentration increased in their experiments under both freshwater and saline environments, indicating that As-rich mineral dissolution becomes dominant in such cases. Arsenic mobilization mechanisms should therefore be treated as a site-specific issue, with risk analyses of target GCS sites guided by preliminary assays of As content of both reservoir and USDW strata.

## Materials and Methods

An integrated framework of systematically combined batch experiments and reactive transport simulations has been developed for better understanding As mobilization mechanisms with CO_2_ leakage into shallow aquifers. The sediment samples for batch experiments are collected from the Chimayo site, a natural CO_2_ analog located at the eastern margin of Espanola basin in northern New Mexico^13^. The shallow drinking water aquifer at this site has been investigated by a series of studies^25, 29, 43^, and water chemistry was analyzed to evaluate mobilizations of trace metals. Observed data show evidence of upward migration of CO_2_ and saline water along the faults, whereas the locations far from the faults are not impacted by CO_2_ or brine. This site provides an example for analyzing the impacts of CO_2_/brine leaked from a GCS site on overlying USDW quality.

### Batch Experiments

**Figure 4:**
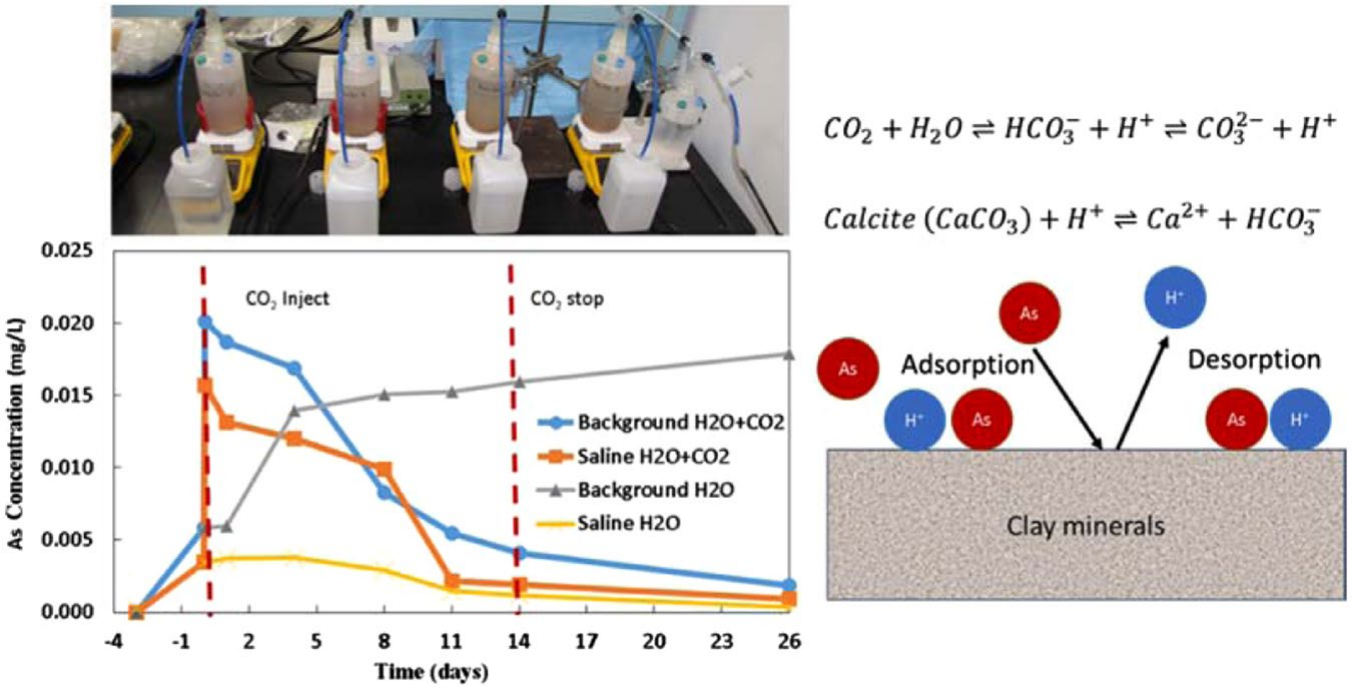
Batch experiments shown arsenic mobilization due to the reactions between the sediments and introduced CO_2_.

Due to high As concentrations in the Chimayo groundwater exposed to CO_2_ and saline water, and the relatively high amount of As present in the sediment samples (up to 147 mg/kg), the behavior of As caused by CO_2_ and saline water intrusion is of specific interest. Batch experiments were conducted to mimic the influx of CO_2_ and saline water into the aquifer in order to evaluate As mobilization due to the reactions between the sediments and CO_2_ (Fig. 4). Details of the experiments were described by Viswanathan *et al*.^25^. Two sets of experiments were conducted, with “background” (BG, low salinity, TDS ~200 mg/L) and “saline” (S, high salinity, TDS ~4000 mg/L) synthetic groundwater, to represent the conditions with/without saline water intrusion. The major components of each synthetic groundwater are listed in Table 2. With each set of experiments, one reactor was exposed to CO_2_ (maintained 1 atm) and one reactor was kept as a steady-state, CO_2_-free control. Before CO_2_ injection, the sediment samples were exposed to the synthetic groundwater for ~3 days to reach a steady state. Water samples were then collected over a 26-day experimental period: 14 days for CO_2_ injection and 12 days for post injection. Major ion and As concentrations were analyzed subsequently.

**Table 2:** Concentrations for major ions of the background and saline batch experiments (mg/L).

Element	Background	Saline	Element	Background	Saline
Ca	5.2	222.1	Cl	28.0	2050.0
Fe	1.4 × 10^−3^	2.2 × 10^−2^	SO4	56.0	333.0
K	0.7	38.6	Si	<DL	<DL
Mg	2.8	220.4	As	<DL	1.5 × 10^−4^
Na	73.9	783.9	pH (unitless)	8.5	6.4

### Geochemical Modeling Approach

In this study, a reactive transport model considering aqueous species complexation, mineral dissolution/precipitation, adsorption/desorption, and cation exchanges was created, in order to evaluate the mechanisms of As mobilization. All simulations were performed with TOUGHREACT V2 and ECO2N^44, 45^.

A total of 54 aqueous complexes that have high impacts on the results were selected for the simulations. The aqueous complexes and their equilibrium constants are listed in Table 3. For all the batch experiments, the reactors were exposed to an oxygen-rich environment (atmosphere). Under this condition, As (V) is considered a dominant form in the aqueous phase, which also corresponds to the conditions of the site (pe > 4). Precipitation/dissolution reactions for all minerals detected in the sample (26% quartz, 3.6% K-feldspar, 2.4% albite, 2.4% anorthite, 0.4% calcite, 0.4% hematite, 43.4% illite, 1.5% kaolinite, and 20.2% smectite) were included in the model. Precipitation of possible secondary minerals was allowed to constrain major ion chemistry in the system. The reaction rates and surface areas of the primary minerals were treated as uncertainty parameters. Although it is hard to detect trace As-bearing minerals (such as As-carbonate minerals) by XRD or SEM-EDS analysis, it is possible that As-rich minerals exist in the sediments^12^ and impact As concentration in the aqueous phase. In this study, the initial amount of trace As minerals were treated as uncertainty parameters.

**Table 3:** Aqueous complexes and their equilibrium constants at 25 °C (Primary species include: H_2_O, H^+^, Ca^2+^, Mg^2+^, Na^+^, K^+^, Fe^2+^, AlO_2_^−^, SiO_2_ (aq), HCO_3_^−^, SO_4_^2−^, Cl^−^, O_2_ (aq), Pb^2+^, H_3_AsO_4_ (aq), UO_2_^2+^).

Species	LogK	Species	LogK	Species	LogK	Species	LogK
OH^−^	13.99	CaCO_3_ (aq)	7.01	SO_2_ (aq)	37.57	HAsO_4_^2−^	9.01
CaCl^+^	0.70	KCl (aq)	1.50	HSO_3_^−^	39.42	AsO_4_^3−^	20.6
CaCl_2_ (aq)	0.65	MgCl^+^	0.14	PbCl^+^	−1.45	HAsO_2_ (aq)	23.54
CaSO_4_ (aq)	−2.10	MgSO_4_ (aq)	−2.38	PbCl_2_ (aq)	−2.01	H_3_AsO_3_ (aq)	23.61
NaCl (aq)	0.78	NaSO_4_^−^	−0.81	PbCl_3_^−^	−1.70	H_2_AsO_3_^−^	32.78
FeCl^+^	0.17	KSO_4_^−^	−0.88	PbCl_4_^2−^	−1.50	UO_2_(CO_3_)_3_^4−^	9.15
FeHCO_3_^+^	−2.04	NaHSiO_3_ (aq)	8.30	PbOH^+^	7.57	UO_2_(CO_3_)_2_^2−^	4.05
FeCO_3_ (aq)	4.88	CaOH^+^	12.85	Pb(OH)_2_ (aq)	17.07	UO_2_(SO_4_)_2_^2−^	−3.97
FeCl_4_^2−^	1.94	NaOH (aq)	14.15	Pb(OH)_3_^−^	28.07	UO_2_Cl^+^	−0.15
NaHCO_3_ (aq)	−0.17	NaCO_3_^−^	9.82	Pb(CO_3_)_2_^2−^	11.24	UO_2_SO_4_ (aq)	−3.06
CaHCO_3_^+^	−1.04	H_3_SiO_4_^−^	9.81	PbO (aq)	16.98	UO_2_OH^+^	5.22
MgHCO_3_^+^	−1.03	Fe^3+^	−8.49	PbHCO_3_^+^	−2.89	UO_2_CO_3_ (aq)	0.39
CO_2_ (aq)	−6.34	CH_4_ (aq)	144.15	PbCO_3_ (aq)	3.06		
CO_3_^2−^	10.33	H_2_ (aq)	46.11	H_2_AsO_4_^−^	2.25		

Adsorption/desorption of As from clay/Fe-rich mineral surfaces was considered as an important process for As mobilization with CO_2_ and saline water intrusion. Hematite, kaolinite, illite and smectite were chosen as principal adsorbents, because they were relatively abundant in the sediment samples and also widely reported by former studies^12, 27, 46^. Arsenic aqueous species HAsO_4_^2−^ and H_2_AsO_4_^−^ were chosen as major surface adsorption ions^41^. Adsorption/desorption reactions are controlled by the total amount of reactive sites (product of amount of adsorbent, site density and adsorbent surface area)^20^, which has high uncertainty for different samples^41^. The local equilibrium of adsorbed As is affected by salinity and pH^37, 38, 46^. Therefore, adsorbent surface area and surface complexation equilibrium constant were selected as uncertainty parameters.

Cation exchange reactions were also considered in the model for major cations, which might affect the response of trace metals and pH to CO_2_ and saline water intrusion. The Gaines-Thomas convention was used in this study^47^. The site-specific parameters of cation selectivity coefficient and cation exchange capacity (CEC) were not measured for this site, and they were treated as uncertainty parameters.

To obtain the best estimations for the uncertainty model parameters, the nonlinear parameter estimation program PEST^48^ was applied. The 26-day experimental data were used to estimate the uncertainty parameters listed above via inversion by minimizing the objective function ***J*** ^48–50^:

5 $${\boldsymbol{J}}=min\sum _{i=1}^{N}{E}_{i}(p);\,{E}_{i}(p)=\sum _{l=1}^{{L}_{i}}{w}_{li}^{2}{({u}_{l}^{i}(p)-{\tilde{u}}_{l}^{i})}^{2}$$

where *E*_*i*_ *(p)* is the sub-objective function from chemical species *i*, *N* (=9) is the number of chemical species, *w*_*ii*_ is the weighting coefficient for the *l*th measurement of the *i*th species, which is computed with the inverse of the standard deviation of the experimental data^49^, and *u*_*l*_^*i*^ and *ũ*_*l*_^*i*^ are the simulated and observed concentrations of Ca, Mg, K, Na, Si, Cl, SO_4_, As and pH. A composite sensitivity analysis of the uncertainty parameters was also conducted in order to determine the most sensitive parameters^48^.
